# Workhouse Waste

**Published:** 1920-12-04

**Authors:** 


### Workhouse Waste.

In *The Nineteenth Century and After* Miss Edith Sellers, who has studied the problems of poverty in almost every country in Europe, gives some instructive figures. Even before the war the cost of an indoor pauper was £38 per annum in London, while last year the cost was in one workhouse £65, in another £77, and in yet another £95 11s. per head yearly. The course advocated by this lady would speedily result in half the population demanding £65 per annum as outdoor relief. She argues that in many cases an amount of outdoor relief, small in comparison with indoor cost, would enable old people to live either with their chidren or in a humble home of their own, and would certainly maintain fatherless children with their mothers, whereas at present the former are often taken away to be educated in expensive schools, where separation inevitably loosens the family tie of affection which it is most desirable to preserve.

